# Supplementary material for: The jingle fallacy in comprehension tests for reading
Source: PLoS One. 2024 Jul 5;19(7):e0306466. doi: 10.1371/journal.pone.0306466 (PMC11226005; doi:10.1371/journal.pone.0306466)
Supplement: Attachment — Submitted filename: Response to Reviewers.docx [file pone.0306466.s001.docx]

Dear Dr Flor,

Before responding to the issues raised by yourself and the reviewers, I would like to thank you all on behalf of myself and my co-authors for the very helpful comments on the previous version of this paper.

We believe we have now successfully addressed the issues raised by yourself and the reviewers. We highlighted the changes in the manuscript in yellow so you can quickly see what has changed.

We look forward to hearing from you soon.

Kind regards,

Charlotte Lee

Comments from the Editor

1.

Lines 285-287:

About the 9 participants that were removed "due poor overall accuracy"

 - accuracy on what? the reading tests? the experimental data?

**This refers to poor accuracy on the comprehension questions during the eye-tracking experiment, we have added this clarification on line 284.**

2.

Lines 321-323.

The high- and low- comprehension demand questions for the experiment had different averages, 23.71 vs. 19.97.
Although the difference is statistically significant, the  difference is less than 4 points on a 100-point scale and very close to the lower (easier) part. It is not quite convincing that the high-comprehension-demand questions were really more difficult. At least some discussion of this is needed.

**This is a valid point and was raised by Reviewer 1 as well. We have now amended our description of the conditions throughout the manuscript to ‘higher and lower comprehension demands’ instead of high and low as suggested by Reviewer 1.**

3.

Lines 370-376: the correlation of NDRT with WIAT-II was just r=0.22.

Doesn't the low correlation (over same subjects) indicate that the test are not measuring the same thing?

Some discussion around this is needed. Are you aware of any other studies that correlated results on those tests?

If the tests are indeed uncorrelated or low-correlated, the 'jingle fallacy' would be shown without the need for eye-tracking studies.

**We have shown in our previous work (Lee et al. submitted) that these tests were weakly correlated (mentioned in line 66) and are not aware of any other direct comparisons in the literature. Indeed, the low correlation between these tests suggests that the tests might not measure the same construct. However, the low correlation is uninformative regarding which of the two measures (or indeed if any) pick up comprehension difficulties (as reflected in eye movements).**

**With the addition of eye-tracking methodology we can further examine where these discrepancies lie – e.g., they may each measure a different aspect of comprehension seen in early or late eye movement measures. This issue is discussed in line 75 “The aim of the current paper …”.**

4.

The notion of high and low scorers is used (line 417), but there is no description on how participants were assigned to such groups for each reading test.  The high- low- scorers implies a dichotomous split.
Consider presenting a scatter plot of WIAT vs NDRT scores - does the distribution of scores on each support a dichotomous split on each test - or maybe more than 2 groups should be considered for each?

**Throughout all the analyses reported in the paper, the WIAT and NDRT scores were treated as continuous predictors. Our graphics aid the interpretation of the continuous effects by the data with separate panels for mean +/- 1SD for each reading test. We have now included a note about this on line 429.**

5.

Given the study by Coleman et al., cited  on lines 96-98, that  college students could answer the
 questions on NDRT comprehension tests and achieve a greater-than-chance level of accuracy
 without actually reading the associated passages - why do you still recommend NDRT as a reading test - rather than a general knowledge test?  Some discussion is needed.

**This is again a very good point and we are happy to add a clarification. Greater than chance accuracy was achieved by participants in the Coleman et al study when responding to questions without actually reading the passages but accuracy was at 44-47% whereas chance level was at 20%. Clearly the scores were well below ceiling level and the test is testing more than just general knowledge. We clarified this on line 98.**

6.

Regarding the study of Eason et al., cited on lines 98-102.

First, you should note that they studied a different test (not NDRT). How that relates to 'cognitive skills' that might underly WIAT or NDRT is unclear.

Moreover, your own citing mentions "when texts and questions were complex". But the text and questions in your study were not complex (see point 3 above). So how does the Eason study  fit here?

**We see how this reference is adding complexity without adding substantial insights. We have removed the reference.**

==============================

Reviewer #1: This study examines the influence of comprehension demands on eye movements as a function of the type of comprehension test. The results suggest that students are generally sensitive to task demands, but there were key differences between skilled and less skilled readers suggesting the existence of a threshold (for less skilled readers). There were also different patterns across the comprehension measures, suggesting they are not interchangeable. Overall, this result is largely consistent with prior research (e.g., Cutting & Scarborough, 2006) that suggests different comprehension measures may tap differential aspects of the construct.
In short, the paper is well written and I commend the authors for pointing out key differences in the two comprehensions tests upfront. Differences in length, genre, administration and production makes these tests more distinct than many other possible comprehension test comparisons. In addition, the review of the eye-tracking research related to this topic is well done and informative. Similarly, the hypotheses are clear and supported by prior research.
That said, the main cautionary note for this study concerns any major claims made between the two comprehension measures (e.g., NDRT is easier or harder than the WIAT, or the low correlation between the two). These are not warranted as the NDRT was not administered with adequate time limits. Please note the non-standardized administration of the NDRT as a limitation.

**We thank the reviewer for their very positive review of our paper.**

**Our choice for a non-standardized administration of the NDRT came with a cost being the reduced comparability with experiments using the standardized administration. However, even though we acknowledge the cost of this choice, we are confident in our choice for the reduced time limit because of a) the demonstrated increased variance within the population which we study, i.e., skilled readers and b) the fact that the shortened version is being used more and more for this group of participants.**

**We write on lines 662 – 669: “We also note that the NDRT was not administered with the standard time limit. A precedent has been set by Andrews et al [51] for administering a shortened version of the NDRT for researchers examining individual differences in skilled readers’ eye movement patterns since it increases the variance between skilled readers. This choice might limit the comparability to research that uses the NDRT with the standard time limit. However, since our focus was on skilled readers and the NDRT shortened time limit is increasing in popularity in the field of examining skilled reading, we feel the choice for the shortened version of the NDRT was justified.“**

Larger issues
The terms low and high comprehension demands are somewhat misleading. I was expecting deeper comprehension questions in the high demand condition. Maybe it would be more accurate to call the conditions “lower” and “higher” as the judgement is relative, not absolute. Similarly, it is not that the questions are deeper per se, but the length and detail of the response required that makes it harder. In other words, “what the passage is about” and “what is the main idea” are not that different in terms of the question alone.

**We have now amended this description of the comprehension demands throughout the manuscript to reflect ‘higher’ and ‘lower’ comprehension demands as suggested by the reviewer. And we also added a clarification of the manipulation on line 315.**

What were the properties of the comprehension questions from the free online practice tests? The limited response options may impact the reliability and subsequently the conclusions.

**We have included more description of how the questions were adapted from online tests in lin****e 315-319. Original questions were the ‘higher demands’ questions, and we created a ‘lower demands alternative for each of them.** **All questions had two options from which participants were required to select an answer. We may have limited capacity to find differences in accuracy due to the limited response options and have now included this as a limitation in line 670. However, this would not limit findings drawn from the eye movement record.**

Pg 14 I was surprised you didn’t ask students to rate the perceived complexity of the paragraph rather than ask about its naturalness (maybe a suggestion for future research). To this point, there doesn’t seem to be any analyses on the naturalness dimension besides the mean. How does one rate how natural passage is? Relative to what criterion? Seems odd.

**The purpose of this pre-screening was exclusively to identify any passages that were outliers in terms of how readable the material was. We have added clarification of this on line 321. Since the exact same texts were presented in both experimental conditions, the materials read by participants did not differ in complexity between conditions. Also, for our analyses, the passages were included in the random effects structure to ensure that findings were not dependent on which text was presented.**

Pg16 Why was the NDRT stopped after 10 minutes? This is does not represent standardized conditions and thus may impact the interpretations across tests. This should be mentioned as a limitation in the limitations section. Maybe one reason the NDRT and the WIAT were not correlated very high was the fact that the NDRT was speeded because of the reduced administration time. In general, many comprehension assessments correlate between .60-.80. .22 seems very low. Pg19 “Fig 1 shows that high scorers on the NDRT comprehension test reduced the number of fixations further into the experimental session”. Were students aware of how much time they had? If so, they could have been rushing as they progressed.

**We addressed the issue of our choice for the reduced administration time for the NDRT when replying to the comments from Reviewer 1. The limitation is addressed in the discussion as is a justification for the choice of the reduced administration time.**

**Participants were not aware of the time limit for the NDRT, so we have no reason to suggest that it promoted speeded reading. We now state this explicitly on line 372. Also, the experimental data where we see a reduced number of fixations is for the texts presented in the eye tracking session where there were no time limits – not during the NDRT which is an “offline” (not eye-tracking) measure.**

Minor issues
The sample is very unbalanced in terms of gender. This should be mentioned in the limitations section.

**We have now noted this limitation on line 659 and also that this imbalance is very typical for psychology undergraduate students.**

Pg 13 it sounds like you programmed the NDRT on your own (Qualtrics). If the publisher reads the article, they may not be too happy if there was no permission to do so.

**Well spotted! We forgot to mention this. Paper copies of the NDRT were purchased from the publisher and voided whenever we ran a participant online. This information has now been included on line 300.**

Pg14 for transparency, it would be helpful to at least name the other tasks given to participants in the experiment that were outside the scope of this paper as the type of task may relate to interference. The timings would also be helpful as it relates to issues such as fatigue.

**This information has now been included on lines 334 and 363.**

Pg15 how are the literal and inferential questions related to the low and high demand tasks? Please provide some clarity.

**This passage on literal and inferential questions refers to the WIAT-II task which was used as an offline (not eye-tracking) measure of reading ability. The high and low comprehension demands were conditions in the eye tracking experimental session. So, they were not related.**

Pg16 again, please name the unrelated tasks (other online tasks) and timings for the second session for transparency. Also what are the reliabilities of the comprehension tests in session 2.

**This information has now been included on lines 334 and 363.**

Reviewer #2:

I really enjoyed reading this well-written manuscript. I only have one question. What is the reliability of the WIAT-II comprehension test (and the NDRT comprehension) calculated from this sample? This is critical information, especially given that the two tests have low correlation. One interpretation is that the two tests are indeed measuring different constructs as the authors propose. However, this still is the possibility that the low correlation is a result of low reliability of one or two of the tests.

**We thank the reviewer for their positive appreciation of our paper.**

**On lines 375-378 we added:**

**“The reliability for the WIAT-II comprehension test was calculated using the split-half reliability with spearman-brown adjustment to be .76. Given the low number of items for the NDRT comprehension test, we calculated reliability using Cronbach’s alpha to be .80.”**

Looking at the WIAT-II comprehension scores, M=110.47 and SD=9.37, if these are standardized scores, this sample is quite high performing on WIAT-II (the WIAT-II population M=100 and SD=15), with this sample's average score .67 SD above the population mean. There might be a ceiling effect on this test, which prevents its predictive power to be fully realized, which could explain the low correlation to NDRT and its non-significant relation to eye-tracking data.

**The scores used in this study are for the WIAT-II comprehension subtest only rather than the entire WIAT-II. The mean for the comprehension subtest in the general population is 103.11 with SD 16.73 according to adult scoring supplement. We also focussed on skilled readers for this experiment, so our sample is from average to very skilled readers, therefore an above average sample on the WIAT-II is fully expected. The notion that we focus on skilled readers is mentioned throughout the paper.**

I also briefly looked at the data file shared by the authors. Is the WIAT-II comprehension scores saved under the column named "WIATTotal"? If so, the data do not match what is reported in the manuscript, which reports that the range is 71-124 but in the data file the range is 84-134.

**The correct column in the datafile for the raw data is ComprehensionStandardScore – these are scores for the comprehension subtest only (the WIATTotal column includes the other reading subtests, we used this for data cleaning – for example removing participants who were below average readers).**
