# Supplementary material for: The jingle fallacy in comprehension tests for reading
Source: PLoS One. 2024 Jul 5;19(7):e0306466. doi: 10.1371/journal.pone.0306466 (PMC11226005; doi:10.1371/journal.pone.0306466)
Supplement: Attachment — Submitted filename: Response to Reviewers.docx [file pone.0306466.s002.docx]

Dear, Dr. Carels

Thank you for taking the time to review this manuscript. My co-authors and I have now addressed reviewers’ comments in the text below and changes are indicated in the manuscript.

We look forward to hearing from you soon.

Kind regards,

Charlotte Lee

Review Comments to the Author

Please use the space provided to explain your answers to the questions above. You may also include additional comments for the author, including concerns about dual publication, research ethics, or publication ethics. (Please upload your review as an attachment if it exceeds 20,000 characters)

Reviewer #1: Thanks for the opportunity to review a revised version of the manuscript entitled “The jingle fallacy in comprehension tests for reading”. The authors have been responsive to the reviews and I have no additional concerns.

We would first like to thank both reviewers for their helpful comments on this manuscript and previous versions.

Reviewer #2: The key argument of this manuscript is that comprehension tests can be measuring different constructs. This argument has been supported by research focusing on the role of decoding and language comprehension (Keenan et al., 2008, <https://doi.org/10.1080/10888430802132279>) or the role of different types of knowledge (Wang et al., 2021, <https://doi.org/10.1016/j.learninstruc.2021.101462>), among other studies. In all these studies, researchers compared the differences of reading comprehension tests by comparing students' behaviors and by correlating these comprehension measures to other measures, and this manuscript applied a similar approach. However, another important piece of information provided by priror research comparing different comprehension tests is the reliability of tests that are compared. We can only compare whether two tests are measuring the same construct after we are certain that both tests are highly and similarly reliable. Two tests, even measuring the same construct, can appear to have zero correlation if one or both of them are unreliable.

In my previous review, I expressed concerns about the reliability of the two reading comprehension tests. I appreciate the authors providing more information. However, the new information provided seems incomplete, and it only makes me more concerned about altnernative explanations for the low correlation between the two comprehension tests. First, for WIAT-II comprehension, the authors only reported adjusted reliability "split-half reliability with spearman-brown adjustment to be .76". What is Cronbach's alpha? What is the reason for reporting "adjusted reliability" rather than the original empirical reliability? The Spearman-Brown formula helps predict the reliability of a test with a different test length. What is the hypothetical test length that is used to calculate this "adjusted" reliabiliy? Please report the unadjusted empirical reliability.

Second, whereas the WIAT-II used split-half reliability (adjusted), the NDRT used Cronbach's alpha. To my understanding, the NDRT test was timed, so that not all students finished all items. How was this dealt with when calculating Cronbach's alpha? What is the split half reliability of NDRT? The reliability information provided by the authors do not reassure me about the possibility that the low correlation of the two tests is mainly a result of low reliability of either or both tests. More transparency is needed.

Response: In response to comments from Reviewer #2, we have reassessed our use of reliability measures in our analyses. The rationale for originally using two different measures for the comprehension tests in our paper was that these were the reliability estimates given in each respective test user manual. It was reasoned that these would be appropriate to calculate to compare reliability in our data with reliability in the norming samples. Following the helpful comments from reviewer #2, we note that by using the WIAT comprehension test solely on adults, the test no longer differs in length (as it would if children took the test) and therefore does not require the spearman-brown adjustment as suggested by the manual. We agree with reviewer #2 that Cronbach’s alpha is therefore more suitable and now report this measure in the manuscript for both the NDRT and the WIAT-II comprehension tests. Reliability for the NDRT comprehension was more difficult to calculate given that participants may complete a different number of items in the given time. We report a Cronbach’s alpha estimate in which missing data have been removed and note that for participants who answer fewer items in the given time, test reliability will be automatically lower. These estimates are discussed in the method section on lines 375 -382 and again in the limitations section on lines 673 -676. Cronbach’s Alpha is still acceptable for both tests (>.60) but does seem to be lower than the reliability values reported in the user manual. This discrepancy is mentioned and discussed in the paper.

Third, using the data provided by the author (thanks again for sharing the data), I found that students' comprehension scores on both tests significantly deviates from normal distribution. There is a lack of students in the middle of the distribution. Most students were near the top, and some students near the bottom. Deviation from normal distribution may distort the estimation of correlations. Furthermore, from the dataset, the correlation between NDRT comprehension and NDRT vocabulary subtests was also surprisingly low, with r = .16. This again makes me worried that the NDRT test might not be reliable for this sample. One would expect much higher correlation between the two subtests of NDRT.

Response: Regarding deviation of the test data from a normal distribution, we compared the original parametric correlation of the two tests (Pearson’s r = 0.22) with non-parametric correlations (Spearman’s ρ = 0.23) and found them to be extremely comparable. We hope this indicates that any deviation from the normal distribution will not have led to a different conclusion.
