# Supplementary material for: The jingle fallacy in comprehension tests for reading
Source: PLoS One. 2024 Jul 5;19(7):e0306466. doi: 10.1371/journal.pone.0306466 (PMC11226005; doi:10.1371/journal.pone.0306466)
Supplement: Attachment — Submitted filename: PLOS ONE Jingle Jangle Review.docx [file pone.0306466.s003.docx]

**Review: The jingle fallacy in comprehension tests for reading**

This manuscript discusses how different comprehension measures may tap into different underlying aspects of comprehension despite sharing the same name. Researchers compare the extent to which the two comprehension measures are predictive of eye tracking data. Both qualitative and quantitative differences between the tests are discussed. The authors do an excellent job of reviewing the literature and framing the argument.

Overall, this study offers a unique contribution to the literature, especially given its use in an adult population of readers. While the Cronbach’s alpha levels were lower than desired, the others acknowledge this and offer a reasonable explanation (lines 378-382; though it would have been nice to have expanded on this). Additionally, the methods and results section were, for the most part, clearly written (though it was unclear to me what a “full random structure” means in terms of multi-level modeling—random slopes? Or random intercepts? Both?). The discussion section was thorough and addressed some of limitations associated with the study.

I feel the previous reviewers have helped the authors address most major issues. I have no major concerns with the paper and recommend it for publication.
