# Supplementary material for: The jingle fallacy in comprehension tests for reading
Source: PLoS One. 2024 Jul 5;19(7):e0306466. doi: 10.1371/journal.pone.0306466 (PMC11226005; doi:10.1371/journal.pone.0306466)
Supplement: Attachment — Submitted filename: Response to reviewers - P3.docx [file pone.0306466.s004.docx]

Dear Dr Wang,

We would like to thank you and the reviewers for taking the time to review this manuscript. My co-authors and I have now addressed the comments in the text below and changes are indicated in the manuscript.

We look forward to hearing from you soon.

Kind regards,

Dr Charlotte Lee

Journal Requirements:

Please review your reference list to ensure that it is complete and correct. If you have cited papers that have been retracted, please include the rationale for doing so in the manuscript text, or remove these references and replace them with relevant current references. Any changes to the reference list should be mentioned in the rebuttal letter that accompanies your revised manuscript. If you need to cite a retracted article, indicate the article’s retracted status in the References list and also include a citation and full reference for the retraction notice.

Additional Editor Comments:

I enjoyed reading this revised manuscript. I was originally “Reviewer 2”. In Feb 2024, the journal invited me to serve as the Guest Editor to handle this revised manuscript. Since then, I sent out the manuscript to multiple reviewers, and have recently received one review back. The review is provided under “Reviewer 3”.
The authors have provided the necessary information I asked in my previous review. I’d like the authors address the following issues before I can make a recommendation.

First, based on my own reading and Reviewer 3’s comments, it is critical that the authors explicitly acknowledge the potential impact of low reliability of WIAT-II (alpha = .62, barely acceptable) on conclusions. WIAT-II’s low reliability could well be the cause of 1) low correlation to NDRT, and 2) its failure to predict eye-tracking results. These alternative explanations need to be clearly discussed. The disattenuated correlation between WIAT-II and NDRT is actually not bad (.22/sqrt(.62*.75) = .54, which is much stronger, and closer to what one would expect to see as the strength of correlation between two reading comprehension tests. The argument of this paper -- reading comprehension tests can test different things – sets a much higher bar for what might be considered acceptable reliability of a test. Only acknowledging the relatively lower reliability of the tests in lines 673-676 is insufficient.

Second, please add correlation coefficients “overall accuracy on comprehension questions” (line 385) and both WIAT-II and NDRT scores. The former may be lower, due to WIAT-II’s low reliability.

Third, the introduction is nicely written. Adding subheadings between transition of different topics will be very helpful. Also related to subheading, line 384 “Data cleaning” should be moved to line 392, since the first paragraph in Results is not really data cleaning.

Fourth, lines 421-425, please add a citation and rationale for this approach. Intuitively, one would build the simplest model and add more effects to it until new effects are no longer significant.

Author response:

1. We have now included further discussion of how low reliability may impact the interpretation of the results on lines 670-677. We also mention this in the abstract.
2. Correlation coefficients for accuracy on the eye tracking task and WIAT-II and NDRT comprehension scores have now been included on lines 396 - 398.
3. We have now added subheadings in the discussion so that the different topics are clearly signposted. We have also moved the Data Cleaning subheading to line 399.
4. We have now moved the citation for the model trimming procedure to the end of the sentence on lines 425 - 433 so that it is more clearly signposted and we have now included some clearer explanation of the model building/trimming method used.

Review Comments to the Author

Please use the space provided to explain your answers to the questions above. You may also include additional comments for the author, including concerns about dual publication, research ethics, or publication ethics. (Please upload your review as an attachment if it exceeds 20,000 characters)

Reviewer #3: This manuscript discusses how different comprehension measures may tap into different underlying aspects of comprehension despite sharing the same name. Researchers compare the extent to which the two comprehension measures are predictive of eye tracking data. Both qualitative and quantitative differences between the tests are discussed. The authors do an excellent job of reviewing the literature and framing the argument.

Overall, this study offers a unique contribution to the literature, especially given its use in an adult population of readers. While the Cronbach’s alpha levels were lower than desired, the others acknowledge this and offer a reasonable explanation (lines 378-382; though it would have been nice to have expanded on this).

Additionally, the methods and results section were, for the most part, clearly written (though it was unclear to me what a “full random structure” means in terms of multi-level modeling—random slopes? Or random intercepts? Both?). The discussion section was thorough and addressed some of limitations associated with the study.
I feel the previous reviewers have helped the authors address most major issues. I have no major concerns with the paper and recommend it for publication.

Author response:

1. We thank you for this comment and have now added further discussion of the low alpha for the WIAT-II on lines 670-677.
2. We have also added further explanation for the structure and trimming procedure for the LMMs on lines 425-433.
